# The Moss *Physcomitrella patens* Is Hyperresistant to DNA Double-Strand Breaks Induced by γ-Irradiation

**DOI:** 10.3390/genes9020076

**Published:** 2018-02-07

**Authors:** Yuichiro Yokota, Ayako N. Sakamoto

**Affiliations:** Department of Radiation-Applied Biology Research, Takasaki Advanced Radiation Research Institute (TARRI), National Institutes for Quantum and Radiological Science and Technology (QST), Watanuki-machi 1233, Takasaki, Gunma 370-1292, Japan; sakamoto.ayako@qst.go.jp

**Keywords:** moss, *Physcomitrella patens*, protoplasts, γ-rays, radiosensitivity, DNA double-strand breaks, pulsed-field gel electrophoresis

## Abstract

The purpose of this study was to investigate whether the moss *Physcomitrella patens* cells are more resistant to ionizing radiation than animal cells. Protoplasts derived from *P. patens* protonemata were irradiated with γ-rays of 50–1000 gray (Gy). Clonogenicity of the protoplasts decreased in a γ-ray dose-dependent manner. The dose that decreased clonogenicity by half (LD_50_) was 277 Gy, which indicated that the moss protoplasts were 200-times more radioresistant than human cells. To investigate the mechanism of radioresistance in *P. patens*, we irradiated protoplasts on ice and initial double-strand break (DSB) yields were measured using the pulsed-field gel electrophoresis assay. Induced DSBs linearly increased dependent on the γ-ray dose and the DSB yield per Gb DNA per Gy was 2.2. The DSB yield in *P. patens* was half to one-third of those reported in mammals and yeasts, indicating that DSBs are difficult to induce in *P. patens*. The DSB yield per cell per LD_50_ dose in *P. patens* was 311, which is three- to six-times higher than those in mammals and yeasts, implying that *P. patens* is hyperresistant to DSBs. *Physcomitrella patens* is indicated to possess unique mechanisms to inhibit DSB induction and provide resistance to high numbers of DSBs.

## 1. Introduction

Organisms show diverse radiosensitivity in a broad range [1,2] and are thus expected to possess various genome maintenance strategies. Terrestrial plants are often hyperresistant to ionizing radiation, although the cells contain relatively large contents of genomic DNA, which is the most important biological target of radiation. After *Arabidopsis thaliana* and tobacco (*Nicotiana tabacum*) dry seeds were irradiated with electron radiation or γ-rays and seedlings grown to measure survival rates, the doses that decreased the survival rate by half (LD_50_) were about 1250 and 700 gray (Gy), respectively [3,4]. However, few studies have assessed the radiosensitivity of single plant cells and thus it remains unclear whether plants are equipped with hyperresistance to radiation at the cellular level. In a previous study, we investigated the clonogenicity of protoplasts derived from the tobacco BY-2 cell line and determined that tobacco protoplasts were 10-times more resistant to radiation than mammalian cells [5].

Ionizing radiation induces various types of DNA damage [6]. Among the types of damage, double-strand breaks (DSBs) are difficult to repair accurately and the impacts are the most serious biologically. To study the mechanism of induction and/or repair of DSBs, several techniques have been developed to detect DSBs [7]. Pulsed-field gel electrophoresis (PFGE) is one of the quantitative methods developed. We previously performed a comparative analysis of initial DSB yields between tobacco protoplasts and Chinese hamster CHO-K1 cells, and reported that the yield per Gb DNA per Gy (Gb DNA^−1^ Gy^−1^) in tobacco was only one-third of that induced in the Chinese hamster [8]. Friesner et al. [9] measured DSBs in *A. thaliana* using an antibody to phosphorylated H2A histone family, member X (γ-H2AX), a biological marker of DSBs, and reported that the DSB yield in *A. thaliana* was similar to that in tobacco and less than those in mammals. Furthermore, at least two-times more DSBs are necessary to inactivate tobacco and *A. thaliana* cells compared with mammalian cells [8,9]. However, the mechanism of plant hyperresistance to DSBs is unknown.

The ultimate goal of our research is to elucidate the mechanisms of plant cell hyperresistance to ionizing radiation. The moss *Physcomitrella patens* is an ideal model terrestrial plant to achieve this objective; a complete genome sequence for *P. patens* is available [10], protoplasts are readily isolated and cultured [11], and gene targeting is feasible [12]. The aims of the present study were to analyze the relationship between radiosensitivity and the DNA DSB induction rate of *P. patens* cells and to elucidate the mechanism of hyperresistance to ionizing radiation. Isolated protoplasts were irradiated with γ-rays, the radiosensitivity was evaluated, and the initial DSB yield was quantified. This is the first report that quantifies the initial DSB yield in a moss and compares it with hyperresistance to radiation.

## 2. Materials and Methods

### 2.1. Suspension Culture

A culture of the moss *P. patens* was kindly gifted from Daisuke Takezawa (Saitama University, Saitama, Japan). The protonemata were disrupted with a homogenizer (Microtec Co., Ltd., Chiba, Japan) and suspension-cultured in a basal medium developed specifically for the moss (called as BCDAT) without agar [11], on a reciprocal shaker at 120 rpm and 23 °C under continuous light. The suspension culture was subcultured every week in a 1:5 dilution.

### 2.2. Protoplast Isolation

The suspension-cultured protonemata were collected by centrifugation at 1000× *g* for 5 min. Centrifugation was always performed at 20 °C in the present study. To isolate protoplasts, the cell wall of the collected protonemata was digested in enzyme solution consisting of 0.5% Macerozyme R-200, 1% Cellulase “Onozuka” RS (Yakult Pharmaceutical Industry, Tokyo, Japan), and 8% mannitol, and incubated for 3 h at 23 °C without light. The isolated protoplasts were filtered through a 40-μm-diameter cell strainer (Corning, NY, USA), washed in 8% mannitol solution three times, and collected at 200× *g* for 5 min. 

### 2.3. Analysis of Cell Cycle Phase Distribution

Protoplasts isolated from the protonemata suspension culture were resuspended in nuclei extraction buffer (CyStain UV Precise P, Partec GmbH, Münster, Germany) and incubated for 30 min. Extracted nuclei were filtered through a 30-μm mesh nylon sieve, and stained with four volumes of 4′,6-diamidino-2-phenylindole (DAPI) staining buffer (CyStain UV Precise P, Partec) for 30 min. The cell cycle in *P. patens* protoplasts was analyzed using a flow cytometer (type PA, Partec). As a reference, cell nuclei extracted from rosette leaves of *A. thaliana* ecotype Columbia were stained and analyzed under the same conditions.

### 2.4. γ-Irradiation

The protonemata and protoplasts were irradiated with γ-rays [13]. Irradiation experiments were performed at room temperature in the Cobalt-60 irradiation facility of the Takasaki Advanced Radiation Research Institute (TARRI), National Institutes for Quantum and Radiological Science and Technology (QST), Takasaki, Japan.

### 2.5. Growth Assay

Protonemata were irradiated for 10 min at dose rates of between 10 and 50 Gy/min. Irradiated protonemata were suspension-cultured for one week. The protonemata were collected on paper disks by filtration. The collected protonemata were air-dried overnight, and then the dry weight was measured using a microbalance. The dry weight at day 0 was subtracted from that at day 7 to estimate the growth potential for one week. The increased weight of the irradiated protonemata was normalized by dividing it by that of sham-irradiated protonemata.

### 2.6. Colony Formation Assay

The colony formation assay is a conventional method used to measure the survival rate of single mammalian cells after treatment with abiotic stresses [14], which we applied for measurement of the survival rate of moss protoplasts. 

Isolated protoplasts were resuspended in the protoplast liquid medium [11] and incubated for 24 h at 23 °C without light. After incubation, the protoplasts were collected by centrifugation at 200× *g* for 5 min. The collected protoplasts were resuspended in a protoplast regeneration medium (PRM/T) without agar [11], counted using a hemocytometer, and the density was adjusted to 1000–5000 cells/mL. Protoplasts were irradiated for 10 min at dose rates of between 5 and 45 Gy/min. Immediately after irradiation, the protoplasts were mixed with an equal volume of PRM/T medium supplemented with 1.6% SeaPlaque low-melting agarose (Lonza, Basel, Switzerland). One milliliter of the mixture was dropped and spread on 60-mm-square hydrated cellophane (Bio-Rad Laboratories, Hercules, CA, USA), which was laid on PRM/T medium supplemented with 0.8% agar. The protoplasts were cultured at 23 °C under light for two to three weeks to allow surviving protoplasts to form visible colonies including 50 or more cells. Three to four days after the spreading, the cellophane and protoplasts were moved onto BCDAT medium supplemented with 0.8% agar. The colony formation rate was calculated by dividing the number of colonies by that of inoculated protoplasts. The survival rate was derived by dividing the colony formation rate of irradiated protoplasts by that of sham-irradiated protoplasts.

### 2.7. Pulsed-Field Gel Electrophoresis Assay

Protoplasts were irradiated immediately after isolation for the DSB assay to avoid, if any, an effect of cell wall regeneration. Isolated protoplasts were resuspended in 8% mannitol, counted using a hemocytometer, and the density was adjusted to 2.5 × 10^6^ cells/mL. The protoplast suspension was mixed with an equal volume of 8% mannitol supplemented with 1.5% agarose GB (Nippon Gene, Tokyo, Japan). The mixture was poured into plug molds (80 μL volume per mold; Bio-Rad Laboratories) and solidified at 4 °C. The agarose plugs were placed on ice during irradiation for 20–30 min at dose rates of between 3.3 and 50 Gy/min. Immediately after irradiation, the agarose plugs were incubated in proteinase buffer, which comprised 1 mg/mL proteinase K (Wako, Tokyo, Japan) and 1% sodium N-lauroyl sarcosinate in 0.5 M ethylenediaminetetraacetic acid (EDTA) (pH 8.0), for 1 h at 4 °C and then for 24 h at 50 °C. After incubation, the plugs were washed with 0.5× Tris-borate-EDTA (TBE) buffer for 30 min with gentle agitation; this process was repeated four times. Finally, the plugs were embedded in electrophoresis gel, which contained 1% Pulsed Field Certified™ agarose (Bio-Rad Laboratories) in 0.5× TBE buffer. The pulse-field gel electrophoresis (PFGE) assay was performed with a CHEF DR-III system (Bio-Rad Laboratories) under the following conditions: 2 L of 0.5× TBE buffer was constantly cooled at 14 °C, initial switch time of 60 s, final switch time of 120 s, run time of 24 h, voltage of 6 V/cm, and included angle of 120°. Chromosomal DNA of *Saccaromyces cerevisiae* (Bio-Rad Laboratories) was used as the DNA size standard.

### 2.8. Quantification of Double-Strand Breaks

After PFGE, the electrophoresis gel was dehydrated in a gel dryer (Bio-Rad Laboratories) and rehydrated in distilled water to improve the sensitivity of the measurement of DNA content in the gel [15]. After rehydration, the gel was stained with 0.01% SYBR Green I (Takara Bio, Kusatsu, Japan) in 0.5× TBE buffer at 50 °C for 3 h. The electrophoresed DNA fragments were visualized using an ultraviolet (UV) transilluminator, and recorded as a digital image using a gel imager equipped with an SYBR Green filter (all from ATTO, Tokyo, Japan). The fluorescence intensity of the background lane was subtracted from that of the sample lane. Based on the assumption of random distribution of DSBs on chromosomal DNA, the DSB yield was quantified using the following Equation [16]:

*F_<k_* = 1 − e^−(*rk*/*n*)^ × (1 + *rk*/*n* − *rk*^2^/*n*^2^),

where *F_<k_* is the fraction of DNA fragments shorter than an exclusion size *k*, *n* is the average size of chromosomes (*n* > *k*), and *r* is the number of DSBs per average size of chromosomes. The Phytozome V3.1 assembly detailed 27 chromosomes varying between 30.2 and 5.2 Mb in length with an average of 17.3 Mb. The exclusion size *k* of 1.6 Mb was used in the present study.

## 3. Results

### 3.1. Major Fractions of Protonema Protoplasts Are Haploid and in G*_0_*/G*_1_* Phase

**Figure 1:**
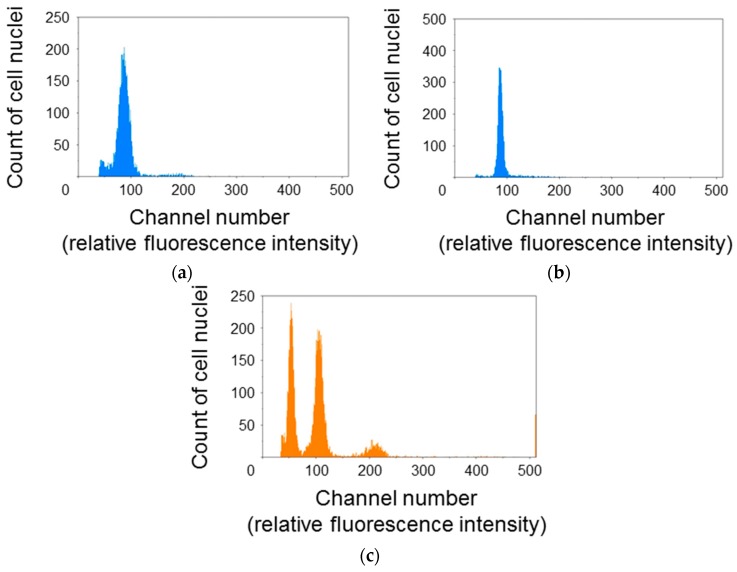
Cell cycle phase distribution of *Physcomitrella patens* protoplasts. Cell nuclei extracted from *P. patens* protoplasts immediately after isolation (**a**); *P. patens* protoplasts incubated for 24 h (**b**); and *Arabidopsis thaliana* rosette leaves, as a reference (**c**); were stained with 4′,6-diamidino-2-phenylindole (DAPI) and the fluorescence intensity was measured using a flow cytometer under the same conditions. Major fractions of *P. patens* protoplasts were presumed to be haploid and in the G_0_/G_1_ phase.

To elucidate the cell cycle phase distribution of protonema protoplasts, the relative fluorescence intensity of DAPI-stained cell nuclei was measured using the flow cytometer (Figure 1). The histogram for *P. patens* protoplasts showed a single peak at around channel 100 (Figure 1a,b). The relative fluorescence intensity for cell nuclei of *A. thaliana* rosette leaves was also measured as a reference. *A. thaliana* cell nuclei showed three peaks at around channels 50, 100, and 200 (Figure 1c). Additional peaks were not detected even when the gain of the flow cytometer was raised or reduced. So, it was decided that the peaks at around channel 100 were 1C moss and 4C *Arabidopsis*. *A. thaliana* is diploid and its 1C genome content is 135 Mb [17]. Thus, the second peak for *A. thaliana* was equivalent to 540 Mb. The 1C genome content of the haploid *P. patens* gametophyte is 511 Mb [18]. Given that the single peak of *P. patens* was close to the second peak of *A. thaliana*, major fractions of *P. patens* protoplasts were presumed to be haploid and in the G_0_/G_1_ phase.

### 3.2. Growth Potential of Protonemata Decreased with Increasing Dose of γ-rays

**Figure 2:**
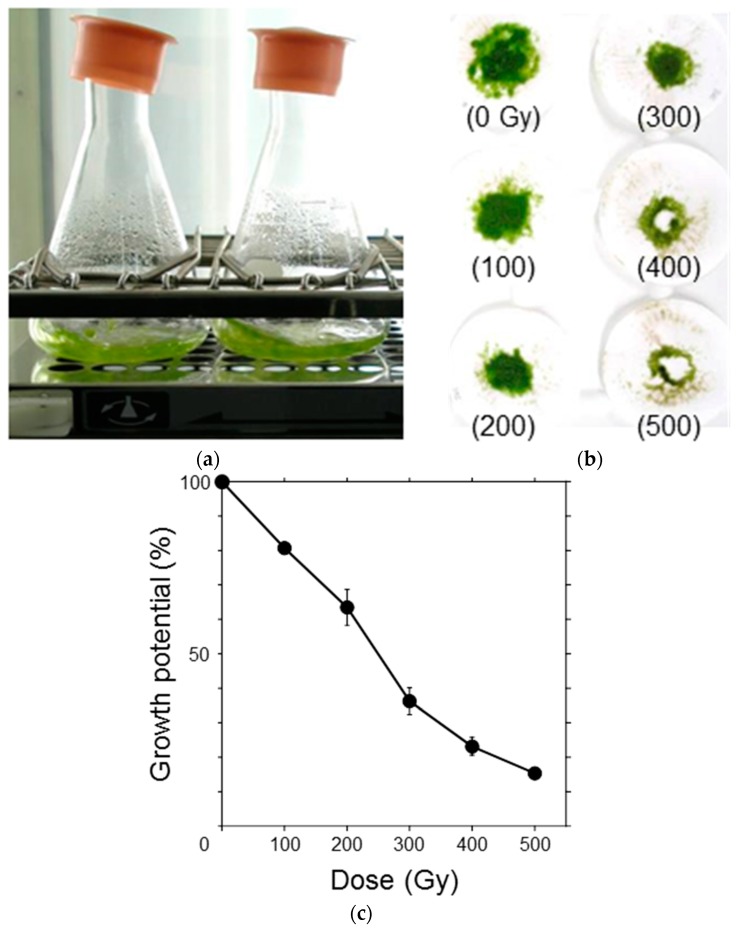
Growth inhibition of *P. patens* protonemata after γ-ray irradiation. (**a**) After irradiation, the protonemata were suspension-cultured for one week under light; (**b**) After incubation, the protonemata were filtered on paper disks, air-dried overnight, and the dry weight was measured. The absorbed doses (in gray; Gy) are specified in parentheses; (**c**) Increase in dry weight over one week was normalized, and the growth potential was plotted as a function of γ-ray dose. The data are the mean ± standard error of the mean (SEM) derived from at least three independent experiments. The growth potential decreased in a dose-dependent manner. The dose that decreased the potential by half was ~250 Gy.

To elucidate the effects of ionizing radiation on the growth potential of *P. patens* protonemata, the protonemata were irradiated with γ-rays in the range of 100–500 Gy and suspension-cultured for one week (Figure 2a). After incubation, the protonemata were filtered on paper disks and the dry weight was measured (Figure 2b). Increase in dry weight, which is a measure of growth potential, of the protonemata for one week, decreased in a dose-dependent manner and the dose that decreased the potential by half was ~250 Gy (Figure 2c). Considering that the moss protonema is haploid and does not possess a duplicate set of chromosomes, the observed radiosensitivity was relatively low.

### 3.3. Survival Rate of Protoplasts Decreased with Increasing γ-ray Dose

**Figure 3:**
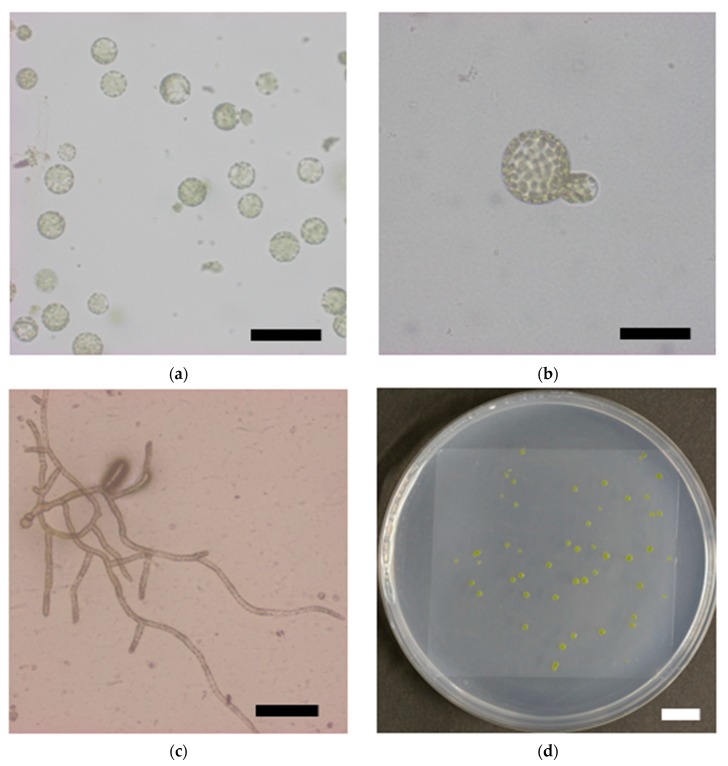

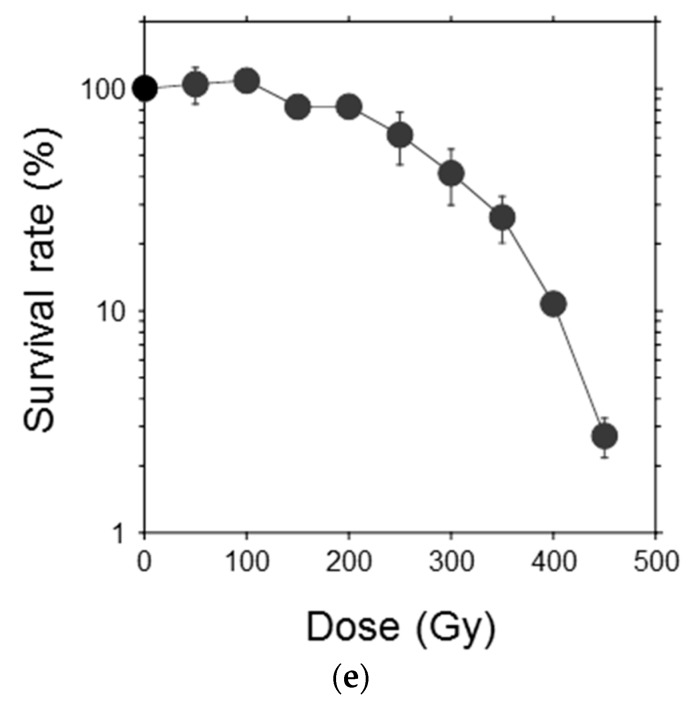
Reduction in survival rate of *P. patens* protoplasts after γ-irradiation. Representative images of (**a**) isolated protoplasts, (**b**) asymmetric first cell division; (**c**) a proliferating colony, and (**d**) visible colonies are shown. Bars indicate 100 μm, 50 μm, 200 μm; and 10 mm in length in (**a**–**d**), respectively; (**e**) Survival rate of protoplasts plotted as a function of γ-ray dose. The data are the mean ± SEM of three or more independent experiments. The survival rate decreased with increasing γ-ray dose. The dose that decreased the survival rate by 50% (LD_50_) was 277 Gy.

To examine the response of single moss cells to ionizing radiation, the protonema protoplasts were irradiated and their clonogenicity was investigated (Figure 3a–d). Survival rate of the protoplasts decreased with increasing γ-ray dose and the LD_50_ was 277 Gy (Figure 3e). The LD_50_ of the protoplasts was similar to the dose that decreased the growth potential of protonemata by half, which indicated that the moss possessed a radioresistance mechanism at the individual cell level.

### 3.4. Double-Strand Break Yields Linearly Increased with Increasing Dose

**Figure 4:**
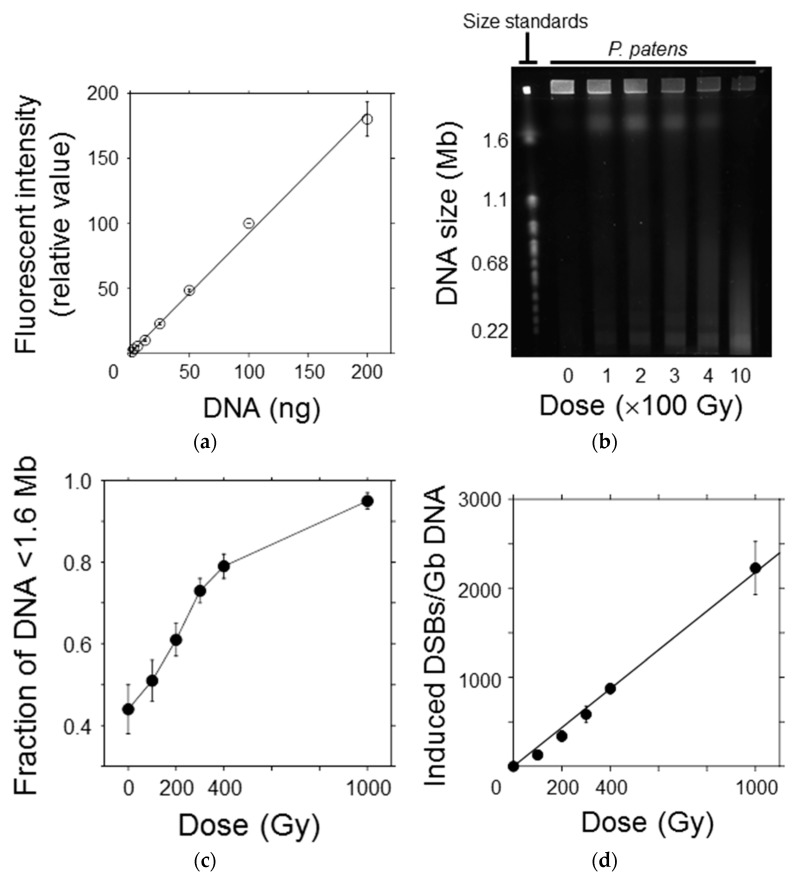
Initial yield of DNA double-strand breaks (DSBs) in *P. patens* protoplasts after γ-irradiation. (**a**) The relationship between DNA quantity and fluorescence intensity of SYBR Green I was almost linear, indicating that DNA fragments could be quantitatively detected in this range; (**b**) A representative image showing the DNA migration pattern in the electrophoresis gel. Chromosomal DNA of *Saccaromyces cerevisiae* was used as the molecular size standard. Chromosomal DNA of the protoplasts was reduced in size with increasing γ-ray dose; (**c**) The fraction of DNA fragments smaller than the exclusion size of 1.6 Mb increased in a dose-dependent manner; (**d**) Number of DSBs induced per Gb DNA plotted as a function of γ-ray dose. The number of DSBs increased linearly with increasing γ-ray dose. The initial DSB yield per Gb DNA per Gy was estimated to be 2.2 from the slope of the regression line.

To elucidate the hyperresistant mechanism of the protonemata and protoplasts to radiation, the initial DSB yield was quantified using the PFGE assay immediately after irradiation. The relationship between DNA quantity and fluorescence intensity of SYBR Green I, which intercalated into double-strand DNA, was linear in the range up to 200 ng per plug size (Figure 4a). Thus, the migration pattern of DNA fragments in the PFGE gel could be quantitatively detected with this system in the study range (Figure 4b). The size of chromosomal DNA decreased with increasing γ-ray dose because of fragmentation by DSB induction. However, DNA of up to 7% was retained inside the agarose plug at higher γ-ray doses. This was probably caused by nuclear proteins that are not easily degraded by the proteinase treatment [8]. The fraction of DNA fragments smaller than the exclusion size of 1.6 Mb (*F*_<1.6Mb_) was normalized by dividing it by 0.93 to avoid underestimating the DSB yield, as reported by Cedervall et al. [19]. The normalized *F*_<1.6Mb_ values increased in a dose-dependent manner (Figure 4c). Using the equation and the average chromosomal size of *P. patens*, described in Section 2.8, *F*_<1.6Mb_ was converted into the number of DSBs induced per Gb DNA (Figure 4d). A linear relationship was observed between the number of induced DSBs and γ-ray dose up to 1000 Gy. From the slope of the regression line, the initial DSB yield was estimated to be 2.2 Gb DNA^−1^ Gy^−1^ (Table 1). The initial DSB yields in tobacco and Chinese hamster cells shown in Table 1 were obtained in our previous study [8]. Because the DSB yield in *P. patens* cells has been quantified with the same protocol as the previous study, those of *P. patens*, tobacco, and Chinese hamster cells are comparable to one another. The number of DSBs induced per cell per LD_50_ was calculated to be 311 by multiplying the DSB yield (Gb DNA^−1^ Gy^−1^) and DNA content (Gb DNA cell^−1^) by LD_50_ (Gy); in other words, 311 DSBs are sufficient to decrease the clonogenicity of the moss protoplasts by half.

## 4. Discussion

Organisms show broad diversity in radiosensitivity [1,2]. The LD_50_ of *P. patens* protoplasts was 277 Gy (Figure 3e), which indicated that the moss protoplasts were 200-times more resistant to ionizing radiation compared with that of human non-cancer cells (1.4 Gy [22]). Considering that genomic DNA content is 511 Mb in haploid *P. patens* cells and 6.0 Gb in diploid human cells, more abundant DNA damages appear to be induced in *P. patens* cells than in human cells after LD_50_ irradiation. The moss is likely more resistant to DNA damage than human cells. *Physcomitrella patens* is unique among plants in its high gene-targeting efficiency, which is due to its high homologous recombination (HR) activity in normal conditions. Kamisugi et al. [23] reported that in bleomycin-treated *P. patens* cells, a large number of genes associated with non-homologous end joining (NHEJ) were up-regulated within 24 h. Therefore, up-regulated NHEJ activity in addition to HR activity might contribute to the radioresistance of *P. patens* cells.

Trouiller et al. [24] and Schaefer et al. [25] reported that the LD_50_ value of wild-type *P. patens* protonema protoplasts was 600 Gy and 800 Gy, respectively. Inconsistency in the results of these authors and the present study may reflect differences in experimental conditions; we initiated culture of the protoplasts immediately post-irradiation, whereas Trouiller et al. [24] and Schaefer et al. [25] incubated the irradiated protoplasts in the dark for 20 h to set the repair time course with the UV-irradiation experiments. In eukaryotic cells, the pre-incubation of irradiated cells under non-proliferative conditions may restore their clonogenicity, and potentially lethal radiation damage may be repaired [26,27]. However, it is not clear at present whether the difference in post-irradiation incubation caused the different LD_50_ values.

It is well known that ionizing radiation induces various types of DNA damage [6]. Among the types of radiation damage, DSBs are believed to be the most difficult to repair accurately and the most lethal DNA damage for most organisms. Several methods, including PFGE [28], γ-H2AX [29], and comet [30] assays, have been developed to detect DSBs. Of these methods, the PFGE assay is a reliable method to quantify DSBs and can quantify DSBs with a similar accuracy as the γ-H2AX assay at high radiation doses [31]. In the present study, to partly elucidate the hyperresistant mechanism of *P. patens* protoplasts to radiation, the initial DSB yield without repairs was quantified using the PFGE assay (Figure 4).

In *P. patens*, the initial DSB yield was 2.2 Gb DNA^−1^ Gy^−1^ (Table 1). In mammals and yeasts, initial DSB yields of 4.2–6.9 have been reported using the PFGE technique (summarized in the review of Prise et al. [7]). In comparison, the initial DSB yield in *P. patens* was only half to one-third those of mammals and yeasts. Some fraction of radiation-induced DSBs can be reduced by scavenging radical species. DeLara et al. [32] reported that half of DSBs were reduced when mammalian V79 cells were irradiated with γ-rays 15 min after the external administration of 0.5 mol/L DMSO. Some types of endogenous protein can play a more important role. Sak et al. [33] reported that in human cancer cells, the removal of histone and other proteins resulted in a 40- to 50-fold increase in DSBs yield. In addition, in the radioresistant tardigrade *Ramazzottius varieornatus*, a novel highly expressed protein may inhibit DSB induction in irradiated cells, and the protein also inhibits DSBs in transgenic human cells [34]. Thus, some unknown proteins or other metabolites may contribute to DNA protection in *P. patens* cells. An additional possibility is that chromatin structure is more condensed in *P. patens* cells than in mammalian cells and yeasts. Newman et al. [35] reported that in Chinese hamster V79 cells, the DSBs yield after X-irradiation was about eight-fold higher in relaxed chromatin than in condensed chromatin. Salicylic acid is the key factor of systemic acquired resistance (SAR). Salicylic acid is biosynthesized after some types of abiotic stresses, including UV-B radiation and reactive oxygen species exposure, and endows plants with stress tolerance via de novo synthesis of antioxidants [36]. When irradiation is long-lasting, SAR likely plays a role to ameliorate its harmful effects. In tobacco BY-2 protoplasts and *A. thaliana* cells, the initial DSB yields of 2.0 and 1.25–2.0 Gb DNA^−1^ Gy^−1^ have been reported [8,9]. These yields are extremely similar to that observed for *P. patens* protoplasts, although the two species are not closely related to mosses. There is likely a common mechanism, by which DSB induction is inhibited biochemically, in terrestrial plants. Identification of candidate molecules that are abundantly present in moss cells or examination of the structure of condensed chromatin might lead to elucidation of the mechanism of plant cell hyperresistance to radiation.

The number of DSBs in *P. patens* (311 DSBs cell^−1^ LD_50_^−1^; Table 1) was calculated by multiplying DSB yield (Gb DNA^−1^ Gy^−1^), DNA content (Gb DNA cell^−1^), and LD_50_ (Gy). The number of DSBs for *P. patens* was about half that of tobacco BY-2 cells, which indicated that the resistance of the moss to DSBs was less than that of tobacco cells. This may at least partly reflect the different ploidy levels: tobacco is allotetraploid and the moss protonema is haploid. Mortimer [37] showed an increase in radioresistance from haploid to diploid yeasts. A direct relationship between ploidy and mean lethal chromosome aberration frequency was reported in human tumor cells exposed to X-rays or γ-rays [38]. Ploidy level in *A. thaliana* can influence sensitivity to UV-B radiation; although accumulated DNA damage in tetraploid cells was expected to be double that in diploid cells, as tetraploid plants were hyperresistant to UV-B compared with diploid plants [39]. Duplicate genomes might contribute to decreased radiosensitivity in plants. Synthesis of mosses of different ploidy levels, which can be achieved through cell fusion, would be useful to investigate the effect of ploidy on hyperresistance to DSBs in mosses.

The number of DSBs cell^−1^ LD_50_^−1^ in the haploid protonemata of *P. patens* was at least three-times higher than that observed in the diploid Chinese hamster and yeasts, which indicates that the moss is hyperresistant to DSBs. *Physcomitrella patens* likely possesses a mechanism that confers resistance to DSBs. In a previous study, we measured DSB rejoining rates using the PFGE assay, and observed almost identical rates of repair between tobacco and Chinese hamster cells [40]. We are currently measuring the DSB rejoining rate in *P. patens* to compare it with other organisms. In addition, whole genome sequencing before and after irradiation will be useful to compare the accuracy of DNA damage repair in *P. patens* and other organisms.

## Figures and Tables

**Table 1:** DNA double-strand break (DSB) yields and radiosensitivity in the moss *P. patens* and other organisms.

Organism	DSB Yield (Gb DNA^−1^ Gy^−1^)	DNA Content (Gb DNA Cell^−1^)	LD_50_ (Gy)	DSB Yield (Cell^−1^ LD_50_^−1^)
Moss	2.2	0.511 [18]	277	311
Tobacco	2.0 [8]	12.3 [8]	27 [5]	664
Chinese hamster	6.6 [8]	6.0 [16]	2.5 [20]	99
Yeast	5.4 [7]	0.024	400 [21]	52
